# Effects of Domiciliary Rehabilitation on the Burden of Care and Quality of Life in Primary Caregivers of Individuals With Spinal Cord Injury

**DOI:** 10.7759/cureus.108456

**Published:** 2026-05-07

**Authors:** Safa Hameed, Shashikant Paswan, Swaleha Furqan, Ranjan Wadhwa

**Affiliations:** 1 Physical Medicine and Rehabilitation, Vardhman Mahavir Medical College and Safdarjung Hospital, New Delhi, IND; 2 Community Medicine, Vardhman Mahavir Medical College and Safdarjung Hospital, New Delhi, IND

**Keywords:** burden of care, domiciliary rehabilitation, physical and rehabilitation medicine, primary caregivers, quality of life, spinal cord injury

## Abstract

Introduction

Caregivers of individuals with spinal cord injury often experience significant physical, emotional, and social challenges that can affect their overall well-being. Understanding how caregiver burden and quality of life change over time, particularly during the transition from inpatient to domiciliary rehabilitation, is essential for developing supportive interventions. This study aimed to examine the course of caregiver burden and quality of life among primary caregivers of patients with spinal cord injury.

Materials and methods

This longitudinal, prospective cohort study was conducted in a tertiary care hospital in a metropolitan city in India. Primary caregivers of patients with spinal cord injury were assessed at two time points: at discharge following the patient's first inpatient rehabilitation (T1) and after three months of domiciliary rehabilitation (T2). Caregiver burden was measured using the Zarit Burden Interview (ZBI), and quality of life was assessed using the WHO Quality of Life-BREF (WHOQOL-BREF) questionnaire.

Results

Domiciliary rehabilitation had a varied impact on caregiver burden. Some caregivers reported a reduction in burden after three months, whereas others experienced a marked increase, including progression to severe burden levels. The overall burden of care before domiciliary rehabilitation (mean ZBI: 41.3, SD: 14.15) and after domiciliary rehabilitation (mean ZBI: 42.46, SD: 19.16) showed no significant difference (p = 0.512), with a mean change of +1.16 (95% CI: -2.40 to 4.72; d = 0.11). Similarly, no significant changes were observed in the physical health (mean change: +0.72; 95% CI: -4.54 to 5.98; d = 0.05; p = 0.783) and psychological health domains (median 50, interquartile range (IQR) 41.6-62.5 at baseline to median 50, IQR 37.5-70.8 at three months; p = 0.856). However, a significant improvement was observed in the social relationships domain of the WHOQOL-BREF (from median 41.67, IQR 33.33-58.33 to median 59.37, IQR 37.49-66.67; p < 0.001), while the environmental domain showed a decline (from mean 51.83, SD 18.16 to mean 45.14, SD: 18.73; mean change: -5.49; 95% CI: -10.00 to -0.99; d = -0.41; p = 0.021) following domiciliary rehabilitation.

Conclusion

The transition from inpatient care to home-based rehabilitation affects caregivers in diverse ways. While some experience improved social support, others face increased challenges within the home environment. These findings highlight the need for tailored caregiver support strategies, including skill-based training, respite care, peer support, and interventions addressing environmental barriers.

## Introduction

Spinal cord injury (SCI) is defined as damage to the spinal cord resulting in loss of motor, sensory, or autonomic function. Globally, an estimated 250,000 to 500,000 individuals sustain SCI each year [1,2]. Timely initiation of rehabilitation is critical for optimal recovery, as delays can adversely affect functional outcomes. SCI is a chronic disabling condition that demands long-term rehabilitation both in hospital and at home. The objectives of rehabilitation are to maintain function, prevent complications such as joint contractures, loss of muscle strength and bone density, pressure ulcers, spasticity, pain, neurogenic bowel and bladder, heterotopic ossification, and depression, and to improve overall quality of life (QoL) [3,4].

The burden of disability following spinal trauma is high, with disability percentages ranging from 57.5% to 100%, accounting for approximately 0.7% of all individuals with disabilities [5]. The transition to home care places a heavy responsibility on caregivers, often family members, who assume physical, emotional, and financial roles without formal compensation. This multidimensional strain is referred to as caregiver burden [6,7].

Most existing studies on caregiver burden and QoL in individuals with SCI are cross-sectional in nature and vary widely in their methodology. As a result, there is limited longitudinal evidence on how caregiver burden and QoL change over time, particularly during the transition from inpatient rehabilitation to home-based care. This phase often brings increased caregiving responsibilities, reduced access to structured support, and added environmental and financial pressures. However, there remains a lack of prospective studies examining caregiver outcomes specifically during the early post-discharge period, which is a critical phase of adaptation for both patients and caregivers.

Domiciliary rehabilitation is provided in the patient’s home environment, allowing care to be tailored to daily living situations and encouraging active involvement of caregivers in the rehabilitation process. In this context, the present prospective longitudinal study was conducted to evaluate the impact of domiciliary rehabilitation on primary caregivers of individuals with SCI. The primary objective was to assess changes in caregiver burden over a three-month period using the Zarit Burden Interview (ZBI), while the secondary objective was to evaluate changes in caregivers’ QoL using the WHO Quality of Life-BREF (WHOQOL-BREF) domains.

Given the limited longitudinal evidence during the early post-discharge phase, this study aimed to explore how caregiver outcomes evolve during this transition, with the expectation that domiciliary rehabilitation may influence caregiver burden and QoL over time.

## Materials and methods

Study design and setting

This prospective cohort study was conducted in the Department of Physical Medicine and Rehabilitation at Vardhman Mahaveer Medical College and Safdarjung Hospital, New Delhi, a tertiary care center located in a metropolitan city in India. The study period extended from March 2023 to September 2024.

Ethical considerations

The study protocol received approval from the Institutional Ethics Committee (approval no: 268/2023). Written informed consent was obtained from all participants prior to enrollment.

Participants

A total of 33 primary caregivers of patients admitted for their first inpatient SCI rehabilitation were recruited for the study, and all participants completed the three-month follow-up assessment. Caregivers were eligible for inclusion if they were aged 18 years or older and provided at least 11 hours of care per day, based on self-reported average daily caregiving time, including assistance with activities of daily living and supervision. Paid caregivers and individuals unlikely to continue caregiving during the domiciliary rehabilitation period, such as those with severe medical illness or pregnancy, were excluded. No participants were lost to follow-up during the study period.

Procedure

The domiciliary rehabilitation program included caregiver education on patient positioning, pressure sore prevention, mobility assistance, bladder and bowel care, and basic physiotherapy exercises such as range-of-motion and positioning techniques. Caregivers received structured training from the rehabilitation team, including physiotherapists under the supervision of a Physical Medicine and Rehabilitation specialist, during the inpatient rehabilitation period prior to discharge.

The program was individualized based on patient needs at discharge, and caregivers were instructed to continue these interventions at home. Accordingly, the frequency and intensity of rehabilitation activities varied across participants depending on individual circumstances and home environments.

At discharge, caregivers were provided with detailed instructions regarding home-based care and were advised to continue these interventions on a daily basis as part of routine caregiving. They were also given contact details of the rehabilitation team to address any queries or concerns during the domiciliary rehabilitation period. Caregivers were instructed to attend monthly follow-up visits at the outpatient rehabilitation clinic, during which adherence to rehabilitation practices was reinforced, medications were reviewed, and discharge advice was reiterated.

In addition, caregivers who had access to smartphones were encouraged to report their daily adherence to rehabilitation activities through a messaging platform; however, this approach had limited uptake due to variability in access to smartphones and internet connectivity, particularly in rural settings.

As the rehabilitation program was individualized, a uniform frequency or intensity of interventions was not predefined. Caregivers were encouraged to integrate rehabilitation activities into daily routines; however, adherence was not formally quantified using standardized measures. Monitoring during the domiciliary phase was primarily based on caregiver self-report during follow-up visits and, where feasible, informal communication with the rehabilitation team. This pragmatic approach reflects real-world clinical practice but may limit strict reproducibility.

At the time of discharge, caregivers completed the ZBI and the WHOQOL-BREF questionnaires to assess caregiver burden and QoL. After a three-month period of domiciliary rehabilitation, caregivers were reassessed using the same instruments to evaluate changes in outcomes.

Sample size calculation

The sample size was estimated based on findings from a previously published longitudinal study conducted among caregivers of individuals with SCI [8]. Although the outcome measures used in that study (Caregiver Strain Index and SF-36) differed from those used in the present study, it was considered an appropriate reference due to the similarities in study design, population, and longitudinal assessment of caregiver burden and QoL.

Assuming a paired comparison design, with a two-sided alpha of 0.05 (corresponding to a 95% confidence level) and 80% power, the minimum required sample size was estimated to be 30 participants based on the variability reported in prior literature.

In addition, a review of patient admissions over the preceding two years (excluding the COVID-19 period) indicated that this sample size was consistent with the average number of eligible cases managed at our center during the study period (March 2023-September 2024), supporting the feasibility of recruitment.

Measurement tools

Zarit Burden Interview

The ZBI consists of 22 items scored from 0 (never) to 4 (almost always), yielding a total score of 0-88. Higher scores indicate greater burden: 0-21: little/no burden; 21-40: mild to moderate; 41-60: moderate to severe; and 61-88: severe burden.

The ZBI is a widely validated instrument with high internal consistency and construct validity across diverse caregiving populations [9,10].

WHOQOL-BREF

This instrument includes 26 items across four domains-physical, psychological, social, and environmental-converted to a 0-100 scale, where higher scores indicate better QoL [11-13].

Data collection

Demographic variables (age, gender, education, occupation, comorbidities, relationship to the patient, family type, and socioeconomic class) and patient details (neurological level of SCI, AIS grade, complications such as pressure ulcers, neurogenic bowel/bladder dysfunction, and neuropathic pain) were recorded.

Statistical analysis

Normality was assessed using the Shapiro-Wilk test. The results indicated that the physical and environmental domains were normally distributed, while the psychological and social domains were not. Accordingly, continuous variables were summarized as mean ± standard deviation (SD) for normally distributed data and median (interquartile range (IQR)) for non-normally distributed data, while categorical variables were presented as frequencies and percentages.

Statistical tests were selected according to data distribution. Parametric tests (paired t-test) were applied to normally distributed variables, while non-parametric tests (Wilcoxon signed-rank test) were used for variables that did not meet normality assumptions. Associations between categorical variables (e.g., caregiver characteristics and burden categories) were analyzed using the chi-square test or Fisher’s exact test, as appropriate. Continuous variables were analyzed using the Mann-Whitney U test or the Kruskal-Wallis test based on data distribution.

A p-value < 0.05 was considered statistically significant. Effect sizes (Cohen’s d for paired samples) and 95% confidence intervals were calculated, where appropriate, to assess the magnitude and clinical relevance of observed changes.

As this was an exploratory study with a limited sample size, adjustments for multiple comparisons were not applied. The analyses were exploratory in nature and intended to identify patterns and generate hypotheses; therefore, the findings should be interpreted with caution.

## Results

Participant characteristics

The mean caregiver age was 38.76 ± 11.53 years (range: 20-55). Most were homemakers (21, 63.6%) and from lower socioeconomic backgrounds (19, 57.6%). The majority (30, 90.9%) lived in nuclear families, and 87.9% were married. Diabetes and hypertension were present in 21.2% and 18.2%, respectively (Table 1).

**Table 1:** Distribution of patients and primary caregivers according to sociodemographic profile

Variable	Patient n (%)	Caregiver n (%)
Age (years)		
<40	23 (69.7%)	16 (48.5%)
≥40	10 (30.3%)	17 (51.5%)
Mean age ± SD	32.79 ± 10.45	38.76 ± 11.53
Range	13-52	20-55
Gender		
Female	9 (27.3%)	27 (81.8%)
Male	24 (72.7%)	6 (18.2%)
Education		
Illiterate	9 (27.3%)	12 (36.4%)
Primary school	11 (33.3%)	12 (36.4%)
Middle school	7 (21.2%)	4 (12.1%)
Secondary school	5 (15.2%)	4 (12.1%)
Graduate	1 (3.0%)	1 (3%)
Occupation		
Homemaker	3 (9.1%)	21 (63.7%)
Student	2 (6.1%)	2 (6.1%)
Unemployed/retired	2 (6.1%)	0 (0%)
Gainfully employed	26 (78.8%)	10 (30.3%)
Socioeconomic status		
Lower	-	19 (57.6%)
Upper lower	-	3 (9.1%)
Lower middle	-	8 (24.2%)
Upper middle	-	1 (3%)
Upper class	-	2 (6.1%)
Type of family		
Joint	-	3 (9.1%)
Nuclear	-	30 (90.9%)

Higher caregiver burden appeared to be associated with lower educational status (p = 0.007), presence of caregiver comorbidities such as diabetes (p = 0.008) and hypertension (p = 0.036), and among caregivers of individuals with cervical-level injuries (p = 0.026).

Correlation analysis revealed a significant negative relationship between caregiver burden and QoL across all domains of the WHOQOL-BREF (p < 0.05).

Among patients, 69.7% had dorsal-level injuries, 24.2% lumbar, and 6.1% cervical. By AIS grade, 69.7% were complete AIS A, 24.2% incomplete AIS B, and 6.1% incomplete AIS C. Pressure ulcers were observed in 36.4%, neurogenic bowel and bladder in 87.9%, and neuropathic pain in 72.2%.

Caregiver burden and quality of life

**Figure 1:**
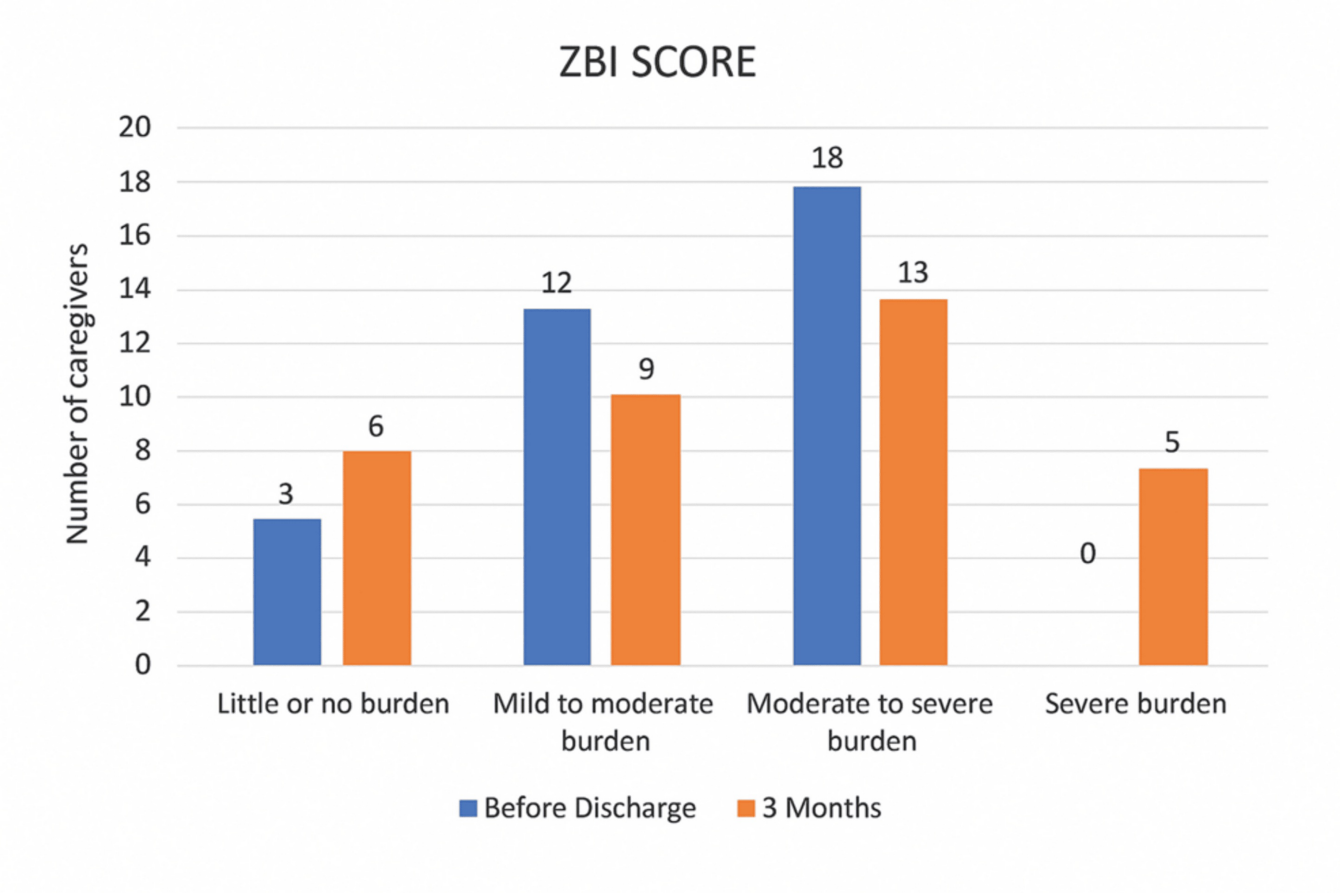
Distribution of primary caregivers according to their ZBI score categories before discharge and after three months of domiciliary rehabilitation ZBI - Zarit Burden Inventory [9,10]

The mean ZBI score changed from 41.30 at discharge to 42.46 at three months, but the change was not statistically significant (p = 0.512) with a mean change of +1.16 (95% CI: -2.40 to 4.72; d = 0.11). Category-wise, the proportion reporting “little/no burden” increased, while the proportion reporting “moderate to severe burden” decreased. However, five out of 33 caregivers newly reported severe burden at follow-up (Table 2, Figure 1).

**Table 2:** Comparison of mean Zarit Burden Interview score before discharge (baseline) and after three months of domiciliary rehabilitation ZBI - Zarit Burden Interview [9,10] The paired t-test was used to test statistical significance. *A p-value of <0.05 was considered statistically significant. Mean difference: +1.16 (95% CI: -2.40 to 4.72; Cohen’s d = 0.11).

	Mean ZBI	SD	Paired t-test
Before discharge	41.3	14.15	t = 0.66, df = 32, p-value = 0.512
Three months	42.46	19.16	

The WHOQOL-BREF domain scores demonstrated mixed changes over the three-month period. There was no significant change in the physical health domain, with mean scores remaining stable from 55.22 ± 16.63 at baseline to 55.94 ± 20.21 at three months (p = 0.783; mean change +0.72, 95% CI: -4.54 to 5.98; d = 0.05). Similarly, the psychological domain showed no significant change, with median scores remaining unchanged at 50 (IQR: 41.6-62.5) at baseline and 50 (IQR: 37.5-70.8) at follow-up (p = 0.856).

In contrast, the social relationships domain showed a significant improvement, with median scores increasing from 41.67 (IQR: 33.33-58.33) to 59.37 (IQR: 37.49-66.67) (p < 0.001). However, the environmental domain demonstrated a significant decline, with mean scores decreasing from 51.83 ± 18.16 to 45.14 ± 18.73 (p = 0.021; mean change -5.49, 95% CI: -10.00 to -0.99; d = -0.41) (Table 3).

**Table 3:** Comparison of WHOQOL-BREF domain scores in caregivers before and after three months of domiciliary rehabilitation WHO Quality of Life-BREF (WHOQOL-BREF) scale [11-13] was used to assess quality of life (QOL) across four domains: physical, psychological, social, and environmental. Data were presented as mean ± standard deviation (SD) for normally distributed variables and median (interquartile range (IQR)) for non-normally distributed variables, based on Shapiro-Wilk test results. Statistical tests were selected based on data distribution assessed using the Shapiro-Wilk test. A paired t-test was used to assess statistical significance for normally distributed data (physical and environmental domains of WHOQOL-BREF), while the Wilcoxon signed-rank test was applied for comparison of non-normally distributed data (psychological and social relationship domains, WHOQOL-BREF). A p-value of <0.05 was taken as significant.

Domain	Baseline score	Score after three months	Test statistic	p-value	Effect size
Physical	55.22 ± 16.63	55.94 ± 20.21	t = 0.28	0.783	d = 0.05
Psychological	50 (41.6-62.5)	50 (37.5-70.8)	Z = 0.18	0.856	-
Social	41.67 (33.33-58.33)	59.37 (37.49-66.67)	Z = 3.60	<0.001	-
Environmental	51.83 ± 18.16	45.14 ± 18.73	t = 2.40	0.021	d = -0.41

Higher burden was associated with lower education (p = 0.007) and caregiver comorbidities such as diabetes (p = 0.008) and hypertension (p = 0.036). Burden was also higher among caregivers of individuals with cervical injuries (p = 0.026). Correlation analysis revealed a significant negative relationship between caregiver burden and QoL across all domains (p < 0.05). The strongest correlations were observed in the psychological domain (r = -0.610, p = 0.035) and the environmental domain (r = -0.550, p = 0.012). Overall, the findings indicate heterogeneous changes in caregiver outcomes rather than a uniform trend.

## Discussion

The demographic profile of individuals with SCI in our study is similar to patterns reported in earlier research. Consistent with previous findings, most individuals with SCI were male, and men were nearly three times more likely than women to sustain such injuries in our cohort. This difference may be related to greater occupational and outdoor exposure among men, who often take on physically demanding work and frequently serve as the primary earners in many households. The mean age of patients in our study (32.79 ± 10.45 years) also reflects trends seen in earlier reports, highlighting the profound socioeconomic impact of SCI, as it commonly affects individuals during the most productive period of their lives (Table 1).

The consequences of injury were particularly challenging for families of lower socioeconomic status, who comprised more than half of our sample. Most patients (78.78%) had been gainfully employed prior to injury, yet only a minority of caregivers were employed, which may contribute to increased financial strain on largely nuclear families. Although joint family systems offered some support, they accounted for only a small proportion of cases (Table 1).

Caregiver characteristics further highlight potential vulnerabilities within the support system. A considerable proportion had low educational attainment and were unemployed, with many serving as parents or spouses of the injured individual. The presence of caregiver comorbidities, such as diabetes and hypertension, may contribute to increased caregiving burden, as also reflected in the observed associations in our study (Table 1).

In this prospective longitudinal study, we examined how caregiver burden and QoL changed among primary caregivers of individuals with SCI during the period of domiciliary rehabilitation. Overall, the ZBI scores did not show a statistically significant change over the three-month follow-up period. However, the individual experiences of caregivers varied. While some reported reduced strain over time, others experienced increased stress, suggesting that caregivers adapt differently to the challenges of home-based care (Table 2, Figure 1).

At baseline, caregivers in our study reported moderate-to-severe levels of burden, which is consistent with findings from recent meta-analyses describing high levels of stress among caregivers of individuals with SCI [9,14]. Previous studies have suggested that structured caregiver support programs may help reduce this burden [7]. In our study, however, the changes observed during the domiciliary rehabilitation period were mixed, and the overall burden levels remained largely unchanged. Notably, some caregivers progressed to severe burden at follow-up, indicating that a subset continued to experience substantial stress despite ongoing rehabilitation support. These findings suggest that additional structured support for caregivers at home may help address the diverse challenges they face.

Although previous studies [15] have suggested that caregiver comorbidities, such as diabetes and hypertension, may increase the burden of care, the associations observed in our study should be interpreted cautiously. While statistically significant relationships were noted between caregiver burden and factors, such as educational status, caregiver comorbidities, and level of injury, these findings are based on exploratory and unadjusted analyses in a relatively small sample, limiting the ability to establish independent effects.

Nevertheless, the presence of these factors among caregivers in our cohort suggests that they may influence caregiving experiences and warrants further investigation in larger studies. Similarly, earlier research, including the findings of Smith et al. [16], has highlighted that caregivers of individuals with cervical-level SCI tend to experience greater burden because these injuries demand more extensive physical assistance and emotional involvement. While statistically significant associations were observed, these findings should be interpreted cautiously as the analyses were exploratory and unadjusted.

The differences in caregiver burden observed in our study likely reflect the complex and multifaceted nature of caregiving. Factors such as the severity of the patient’s condition, family dynamics, coping mechanisms, and the availability of social or practical support can all influence how caregivers experience and manage their responsibilities. The transition to home-based care during domiciliary rehabilitation may interact with these factors in different ways. For some families, structured guidance during rehabilitation may ease caregiving responsibilities, while for others, the demands of home care may become overwhelming when available support is limited. These observations highlight the potential importance of developing individualized caregiver support strategies within community-based rehabilitation programs, especially for those caring for individuals with more severe injuries or managing their own health conditions.

Although the overall QoL scores remained relatively stable during the follow-up period, changes were observed in specific domains. Improvements in the social relationships domain may reflect increased interaction and emotional bonding that can develop when caregivers become actively involved in the rehabilitation process. In contrast, declines in the environmental domain suggest that caregivers may continue to face challenges related to financial strain, accessibility of resources, and the practical demands of home-based care. Similar patterns have been reported by Mohammed et al. [17], emphasizing that caregiver well-being is influenced by both patient-related and environmental factors. These findings suggest that integrating caregiver-focused education and structured follow-up within SCI rehabilitation programs may help address some of these challenges and support caregivers during the transition to home-based care.

The findings of this study highlight the varied and often complex experiences of caregivers during the transition from inpatient to home-based rehabilitation. While some improvement was observed in social relationships, overall caregiver burden remained largely unchanged, and challenges within the home environment appeared to increase. These patterns suggest that domiciliary rehabilitation may influence different dimensions of caregiver well-being in distinct ways. Rather than reflecting a uniform effect, the observed outcomes point to the role of contextual factors such as available support, financial constraints, and the demands of daily care. Together, these findings emphasize the need for individualized and context-sensitive support strategies, particularly within resource-limited settings.

The findings of this study should also be interpreted in the context of the socioeconomic and cultural setting in which it was conducted. In many parts of India, caregiving is predominantly provided by family members, often within nuclear households with limited external support. Cultural expectations around informal caregiving, gender roles, and family responsibility may influence how caregiver burden is perceived and managed. Additionally, the high proportion of caregivers from lower socioeconomic backgrounds in our study may have contributed to the environmental challenges observed during domiciliary rehabilitation. These contextual factors may limit the generalizability of the findings to settings with different healthcare systems, social support structures, or formal caregiving services. Future studies across diverse populations are needed to better understand how these factors influence caregiver outcomes.

Given that most primary outcomes did not show statistically significant change, the findings should be interpreted as exploratory and hypothesis-generating rather than confirmatory.

Limitations

This study has several limitations that should be considered when interpreting the findings. As an observational study, causal inferences regarding the effect of domiciliary rehabilitation on caregiver outcomes cannot be established. The relatively small sample size may limit statistical power and the ability to detect small but clinically meaningful differences, in addition to restricting generalizability due to the single-center design. In addition, the three-month follow-up period provides only a short-term perspective on caregiver burden and QoL and does not capture longer-term trajectories, including potential adaptation, plateau, or worsening of caregiver outcomes over time. As caregiving experiences in SCI are known to evolve over extended periods, the findings of this study should be interpreted as preliminary and reflective of early post-discharge experiences rather than long-term outcomes.

The absence of a control group limits direct comparison across different caregiving contexts. Furthermore, the use of different instruments for sample size estimation and outcome assessment may introduce some methodological inconsistency, although this approach was necessary due to limited comparable longitudinal data. Additionally, the analyses were not adjusted for potential confounding variables, and therefore, independent associations cannot be established.

Response and measurement bias may be present, as caregivers were trained and assessed by the same rehabilitation team, and blinding was not feasible. The exclusion of caregivers with severe medical illness or pregnancy may introduce selection bias, although this was necessary to ensure continuity of caregiving during follow-up. The study did not include a detailed evaluation of specific home-based caregiving tasks, which could have provided additional insight into factors contributing to caregiver strain. Finally, socioeconomic and cultural factors specific to the study population, including reliance on informal family-based caregiving and limited access to external support services, may influence the broader applicability of these findings.

Strengths

Despite these limitations, the study has several notable strengths. The prospective longitudinal design allows for the assessment of changes over time and provides a temporal perspective on caregiver burden and QoL. The use of validated and widely accepted assessment tools, including the ZBI and the WHOQOL-BREF, enhances the reliability of the findings. In addition, the inclusion of both caregiver- and patient-related variables enables a more comprehensive understanding of the caregiving context. The study also focuses on the important transition period from inpatient rehabilitation to home-based care, a phase that is often understudied but critical in shaping caregiver experiences. Furthermore, conducting this study in a tertiary care setting in India offers valuable insights from a resource-constrained healthcare environment, where caregiving responsibilities are often substantial.

## Conclusions

The findings of this study suggest that caregivers experience varied changes in burden and QoL during the period of domiciliary rehabilitation. While overall caregiver burden and most quality-of-life domains did not show statistically significant change over the three-month period, individual experiences differed, with some caregivers reporting reduced strain and others experiencing increased stress. Improvements were observed in social interactions, whereas challenges persisted within the home environment. These findings highlight the complexity of caregiver experiences and suggest that different aspects of caregiver well-being may evolve differently during the transition to home-based care. Supportive measures, such as caregiver education, structured follow-up, and attention to the home environment, may be beneficial and warrant further evaluation in future studies.

Overall, these findings reflect short-term outcomes during the early post-discharge period, and larger longitudinal studies are needed to further clarify these observations and inform clinical practice.
